# Pathogenesis, Diagnostic Pathways, and New Therapeutic and Nutritional Strategies for Pancreatic Cancer-Associated Cachexia

**DOI:** 10.3390/cancers18071060

**Published:** 2026-03-25

**Authors:** Wiktoria Klus, Jagoda Ossowska, Katarzyna Kowalcze, Anna Kiliszczyk, Agnieszka Paziewska

**Affiliations:** 1 Warsaw Genomics S.A., 02-089 Warsaw, Poland; wiktoria.klus@warsawgenomics.pl (W.K.); jagoda.ossowska@warsawgenomics.pl (J.O.); 2 Faculty of Medical and Health Sciences, Institute of Health Sciences, University of Siedlce, 08-110 Siedlce, Poland; katarzyna.kowalcze@uws.edu.pl; 3 Sirene Medical Clinic, 02-796 Warsaw, Poland; 4 Metabolic Diseases Clinic, Damian Medical Center, 02-674 Warsaw, Poland; akiliszczyk@wp.pl

**Keywords:** cachexia, pancreatic cancer, metabolism, microbiome, inflammation, PEI, PERT

## Abstract

**Background/Objectives**: Pancreatic cancer-associated cachexia (CAC) is a complex, multifactorial and multi-organ metabolic syndrome affecting approximately 80% of patients with pancreatic ductal adenocarcinoma (PDAC). Recent epidemiological data show that cachexia is a primary cause of mortality in PDAC, directly accounting for approximately 30% of cancer-related deaths and significantly limiting the tolerability of cancer therapy and is associated with adverse effects of treatment. It is defined by systemic weight loss, skeletal muscle atrophy (sarcopenia), and adipose tissue depletion, often driven by systemic inflammation and metabolic dysregulation. **Methods**: The literature was searched in PubMed and Scopus using combinations of keywords. The search covers the literature between 2016 and 2026, but papers before this period were also included because of their historical importance. Studies with higher evidential value, such as prospective studies, randomized controlled trials, and meta-analyses, were prioritized and emphasized in our analysis. **Results**: CAC in PC arises from a systemic inflammatory response driven by tumor–host interactions and the release of pro-inflammatory mediators, such as growth differentiation factor 15 (GDF-15) and parathyroid hormone-related protein (PTHrP), which promotes anorexia and weight loss. The most commonly used diagnostic criteria include unintentional weight loss of more than 5% of body mass within 6 months, a body mass index (BMI) below 20 kg/m^2^, or weight loss greater than 2% in the presence of sarcopenia. Emerging evidence supports the use of AI-based body composition analysis and novel biomarkers, including GDF-15 levels, to improve the detection and monitoring of cachexia. This review highlights that, despite the absence of pharmacological agents specifically approved for CAC in the United States and Europe, current guidelines recommend multimodal supportive care, including low-dose olanzapine, nutritional support, and exercise-based interventions. Furthermore, we identify recent phase 2 trials targeting the GDF-15 pathway, such as the GDF-15 inhibitor ponsegromab, which have demonstrated significant improvements in body weight and physical activity, suggesting a potential breakthrough in targeted therapies for CAC. **Conclusions**: CAC in PDAC represents a critical unmet medical need in oncology. It manifests as a lethal systemic pathology that demands early identification and targeted personalized pharmacological and nutritional interventions. Early diagnosis and targeted intervention represent promising strategies for improving survival and quality of life in this high-risk patient population.

## Simple Summary

Pancreatic cancer (PC) is one of the deadliest cancers, often detected at advanced stages when patients already suffer from cancer cachexia—a severe loss of body weight, muscle mass and strength. These changes limit the effectiveness of treatment and worsen survival. This review discusses the current diagnostic and therapeutic approaches to cachexia and sarcopenia in PC. By summarizing the available evidence and clinical strategies, it aims to highlight practical ways to improve patient assessment, nutritional support and overall care.

## 1. Introduction

Cachexia, from the Greek “*kakos hexis*”, which means “bad state”, was described for the first time in the early Greek literature, in which it was explained as a consequence of diseases or aging, which led to a lack of physical “conditioning”. In 1906, its symptoms were linked to cancer by Glentworth R. Butler, and in 1915, Howard C. Taylor identified clinical characteristics of cachexia in a range of pathologies [1]. A different name for cachexia is wasting condition, a syndrome caused by many diseases, one of which is cancer, especially an advanced one. Cancer-associated cachexia (CAC) has been defined as a syndrome, characterized by an ongoing loss of skeletal muscle mass, with or without loss of fat tissue, that cannot be fully reversed by conventional muscle nutritional support and leads to progressive functional impairment. It is caused by a combination of reduced food intake and abnormal metabolism [1,2]. It is estimated that it causes the deaths of 2 million people worldwide per year, accounting for approximately 30% of cancer-related deaths. The prevalence of cachexia among patients with cancer varies and depends on its type and stage of disease [1,3,4,5,6,7]. Overall, it is estimated to affect up to 74% of patients with cancer worldwide [3]. Pancreatic cancer is considered a “model” malignancy in cachexia research, as it affects up to 80% of patients with pancreatic cancer, drastically reducing both quality of life (QOL) and the efficacy of oncological therapy [8]. Pancreatic cancer is diagnosed at advanced stages of cancer. No single biomarker currently achieves sufficient sensitivity and specificity for early detection in routine practice. Cachexia is observed in patients even before pancreatic cancer is diagnosed. Metabolic reprogramming in pancreatic tumors is highly energy-intensive and requires significant nutrients, increased tumor metabolism, systemic inflammation, and pharmacotherapy interventions, it contributes tohypercatabolism of pre-cachexia. This feature, along with the physiological changes induced by the tumor, can devastate the patient’s health, negatively impacting prognosis and overall survival (OS). A terminally ill patient’s weight loss can reach 25% of their pre-disease body weight. Due to the frequent occurrence and negative impact on oncological treatments and reduced response to them, it is important to understand how common cachexia syndrome is in patients with cancer [1,2,7].

PC is characterized by rapid progression, early metastasis, and resistance to anticancer therapy. According to the latest data, the survival rate for PC is only 13%, while its incidence is increasing by approximately 1% each year. To prolong the survival of patients with PC and improve their QOL and response to treatment, understanding the pathogenesis of CAC and therapeutic options to help with it is crucial [9,10,11].

In this review, we will collect information regarding the diagnostic pathway and management for CAC.

## 2. Methodology

Due to the narrative nature of the review, no formal assessment of risk of bias was performed; however, studies with higher evidential value, such as prospective studies, randomized controlled trials, and meta-analyses, were prioritized and emphasized in our analysis.

Selection Criteria: The literature was searched in PubMed and Scopus using cachexia- and pancreas-related terms, for example: “cachexia”, “pancreas”, “pancreatic cancer cachexia”, “pancreatic cancer”, “cancer-associated cachexia”, “gut microbiota in cachexia”. This review included original and review articles on cachexia, especially cachexia occurring in patients with pancreatic cancer, published in English in peer-reviewed journals. We excluded case reports, conference abstracts without full text, and publications not providing primary data or up-to-date guidelines. This review covers the literature between 2016 and 2026; several works published before this period are also included because of their historical importance.

## 3. Cancer-Associated Cachexia—Symptoms

CAC is characterized by the severe depletion of lean body mass, including involuntary progressive weight loss and skeletal muscle wasting (sarcopenia), with or without the loss of adipose tissue, and is no longer understood as a nutritional deficiency.

Cachexia cannot be fully reversed by conventional nutritional support alone and leads to progressive functional impairment [1,2,3,4,5,6,12]. PC is associated with mechanical and functional barriers for proper nutrition. Patients with PC experience pain, lack of appetite, fatigue and anorexia, which can lead to anhedonia and prevent the ability to perform daily activities [1,2,4,12]. Cachexia is additionally recognized as a systemic, multi-organ syndrome. Crucially, recent evidence highlights that this tissue loss extends not only to fat or muscle tissue but to vital organs and bones, and specifically manifests as cardiac atrophy (cardiac cachexia), which involves structural remodeling and mass loss of the left ventricle, independent of chemotherapy-induced cardiotoxicity [1,3,13,14].

Cachexia accelerates PC progression, causing a more aggressive clinical course, thus having a negative impact on daily life. Patients with CAC experience numerous complications, including muscle atrophy, cardiac arrhythmia, respiratory weakness, and severe immunosuppression [1,2,4,12]. Other factors such as pain, fever, diarrhea, respiratory difficulties, or psychiatric distress—common during cancer treatment—can further exacerbate appetite loss [7]. They have a higher risk of hospitalization and other complications, which ultimately contribute to a lower OS rate [7,9,15].

Additionally, cachexia leads to mental health complications. CAC causes profound neuropsychiatric symptoms. New research identifies a specific “neuroimmune axis” in which systemic inflammation (e.g., IL-6) directly inhibits dopamine signaling in the brain, causing marked biological apathy, loss of motivation, and fatigue that is distinct from classic depression [4,5,16,17,18,19].

## 4. Cancer-Associated Cachexia—Pathogenesis

CAC involves complex metabolic reprogramming and dysregulation, driven by inflammation and tumor–microenvironment interactions. While the immunological aspects of this wasting condition are not yet fully elucidated, its foundation lies in a chronic inflammatory program triggered by the underlying malignancy [2]. CAC is characterized by reduced food intake and increased resting energy expenditure (REE). These metabolic alterations are driven by a synergistic interplay between tumor-derived catabolic mediators, neuroendocrine stress responses, and persistent systemic inflammation. Furthermore, reduced protein synthesis, insulin resistance, low levels of anabolic hormones, and increased protein breakdown collectively lead to the overall loss of skeletal muscle mass [6,20,21]. Given the variety of malignancies associated with cachexia, it is hypothesized that the multiple systemic effects of the unresolved disease are the root cause of the syndrome. Given the high frequency of cachexia in PC, considerable research has focused on elucidating its mechanisms. This metabolic derailment is uniquely aggressive and leads to profound muscle protein degradation [2,7].

CAC is associated with the release of pro-inflammatory factors [20]. The interaction between cancer cells and healthy tissue—particularly adipose tissue and muscle—drives the observed phenotype [3]. Cancer cells often undergo a metabolic shift from oxidative phosphorylation to cytosolic glycolysis (the Warburg effect), increasing the body’s dependence on glucose. This metabolic reprogramming causes the mobilization of glucose precursors from muscle and adipose tissue, leading to weight loss and muscle atrophy if the process is sustained. Additionally, these changes are mediated by pro-inflammatory cytokines secreted by both tumor cells and immune cells of the organism. Key cytokines include tumor necrosis factor alpha (TNF-α), interferon-gamma (IFN-γ), TGF-β, and several interleukins (IL-6, IL-1β). They stimulate the consumption of lipid stores and muscle degradation and also influence body tissues and tumor microenvironments, causing systemic inflammation, which in combination with malnutrition, leads to cachexia [3,7]. Both immune and non-immune cells release TNF-α, which activates the ubiquitin–proteasome system (UPS), a pathway directly linked to muscle wasting. Elevated levels of TNF-α, IL-1, IL-8, and IL-10 are consistently documented in cachectic patients, resulting in anorexia, muscle atrophy, and increased energy expenditure [20] (Table 1). One example is the research by Sechrist et al., in which the focus was on patients with PC, more precisely PDAC, which is one of the main cancers responsible for cancer-related deaths. According to their study, PDAC cells secrete high levels of IGFBP-3, which has an impact on the development of CAC and the pathologic accumulation of lipids. This suggests that IGFBP-3 should be investigated as a potential therapeutic target for CAC in PDAC [10]. These are not the only studies that found a connection between pancreatic tumor and cachexia. In 2021 Cole et al. published results of their study in which they injected KCKO-luc tumor cells in female mice. After they developed a solid PDAC tumor, the lower limb hind muscles were subjected to histological examination and extraction of RNA. The results of the histology revealed an increase in hematopoietic cells within the muscle of mice and a decrease in the fiber size of some of the muscles in correlation with control mice without the tumor. RNA analysis revealed an increase in the expression of genes like IL-1, IL-6, and TNF. It has also been shown that adipogenic gene expression was increased. All of this suggests that systemic inflammation present because of PDAC leads to inflammatory cell infiltration and to CAC [22]. Although animal models cannot completely mimic the complexity of CAC in humans, they remain an essential tool for elucidating underlying mechanisms and generating hypotheses. Olumoyin et al. conducted research on CAC biomarkers and developed machine-learning models that can use biomarkers from blood to identify pre-cachexia in patients with PDAC. In the study, the level of 35 biomarkers from 184 patients was taken into account; 28 patients were defined as not having cachexia, 53 in the state of pre-cachexia and 103 with CAC [23]. The study published by Park et al. also focused on biomarkers for CAC in patients with PDAC. The criteria for participants were as follows: diagnosed with PDAC, availability of blood sample pre-treatment and ability to determine their status of cachexia. Criteria were met by 202 patients, and their serum was tested for a panel of 42 biomarkers. Based on the research conducted, it was determined that GDF-15 is the best potential “pre-cachexia” biomarker [8].

Recent research indicates the role of the neuroendocrine system, especially the pituitary and adrenal glands and the hypothalamus [7,20,21,24]. The hypothalamus controls appetite and energy expenditure. In the case of the physiological conditions of the body, NPY and AgRP neurons are responsible for stimulating appetite (orexigenic) while POMC and CART neurons suppress it (anorexigenic). In CAC, systemic inflammation disrupts this balance by suppressing NPY/AgRP activity and activating POMC/CART neurons, contributing to anorexia [20,25]. Pro-inflammatory cytokines increase corticotropin-releasing factors, suppress NPY neurons, delay gastric emptying, lower albumin levels and promote lipolysis. Additionally, parathyroid hormone-related protein (PTHrP) reduces food intake, impairing gastric emptying and causing muscle loss [7,20].

Appetite regulation also involves hormones like ghrelin and leptin. Ghrelin, secreted by the stomach, possesses anti-inflammatory properties, prevents muscle degradation, and enhances muscle protein synthesis. For this reason, ghrelin can be a potential therapeutic target in CAC. The second one, leptin, is secreted by adipose tissue and inhibits hunger. Levels of leptin receptors during CAC are increased, which causes anorexia and weight loss [7]. Different catabolic factors stimulated by the tumor that were presented as mediators of metabolic derangement were myostatin and activin [3]. Although cytokine trajectories during active weight loss remain variable, they are known to amplify each other’s effects [1]. In many cancers, a direct correlation exists between cytokine production and cachexia prevalence [7].

**Figure 1:**
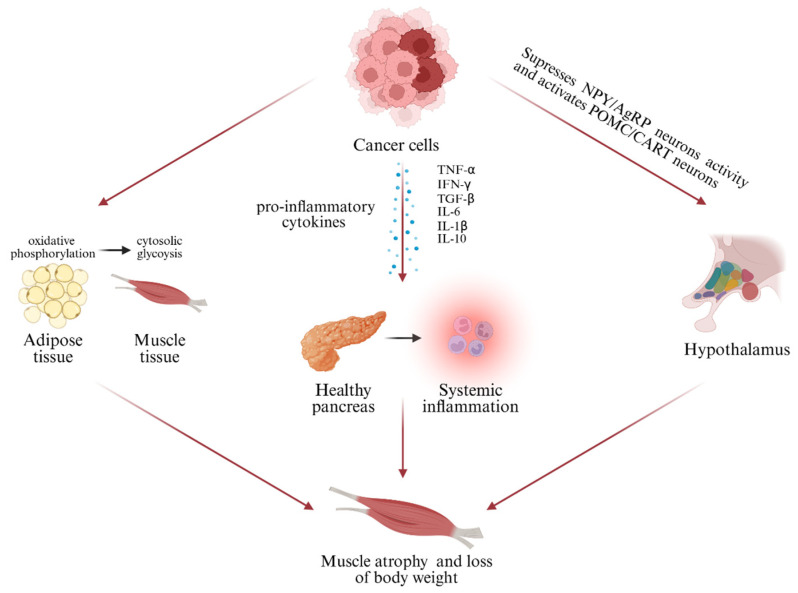
Pathogenesis of CAC. TNF-α: tumor necrosis factor alpha; IFN-γ: interferon-gamma; TGF-β: transforming growth factor beta; IL-6: interleukin 6; IL-1β: interleukin 1 beta; IL-10: interleukin 10; NPY/AgRP neurons: neuropeptide Y/agouti-related peptide neurons; POMC/CART neurons: pro-opiomelanocortin/cocaine- and amphetamine-regulated transcript neurons. Created in Biorender. Paziewska, A. (2026) https://BioRender.com/fb92vcg (accessed on 20 March 2026).

These mediators can be produced directly by damaged cancerous cells or by cells recruited to the microenvironment of the inflammation, like fibroblasts or immune cells, or even by other tissues at the level of the organism. In the case of patients with PC, they are produced by cancerous cells present in the pancreas [1] (Figure 1).

Chronic malabsorption and malnutrition are the result of exocrine pancreatic insufficiency (EPI), a major complication associated with pancreatic cancer and often a serious consequence of surgical resection, caused by reduced or abnormal secretion or activity of pancreatic enzymes. The loss of digestive enzymes leads to severe malabsorption and exacerbation of a negative energy balance. EPI has been associated with abdominal pain, steatorrhea, and unintentional weight loss, as well as clinical symptoms of deficiency of fat-soluble vitamins such as vitamin D. Secondary diabetes (type 3c): Direct destruction of endocrine tissue and insulin resistance contribute to a state of “metabolic starvation” in which cells are unable to use available glucose [16]. All these factors contribute to cachexia in the pancreas and have a negative impact on the prognosis of treatment, contributing to poor tolerance, higher susceptibility to toxicity of cancer treatment and, as a result, more serious side effects and reduced response to anticancer therapies. Metabolic reprogramming linked to cachexia can reduce anti-tumor immunity and the effectiveness of immunotherapy with immune checkpoint inhibitors in patients with cancer, including PD-(L)1 and CTLA-4 inhibitors [15].

Certain therapies induce caloric deficiency by impeding food digestion and absorption; for instance, through anatomical obstructions caused by tumor progression, or malabsorption resulting from surgical resections or treatment side effects. An example is the chemotherapy-related increase in circulating growth differentiation factor 15 (GDF-15) (often seen with platinum-based agents), which induces emesis, nausea, and anorexia [1,26]. GDF-15 interacts with receptor GFRAL (GDNF family receptor alpha-like), which is located in the central nervous system. Preclinical studies using antibodies against GDF-15 and GFRAL have demonstrated potential in counteracting this weight loss [7]. Another example is the use of synthetic glucocorticoids, such as dexamethasone, often prescribed as supportive care (co-medication). Paradoxically, long-term use is responsible for inducing muscle atrophy and dysfunction [1,27]. Furthermore, gastrointestinal dysfunction plays a significant role in CAC development. Cancer treatments contribute to chronic malabsorption, which indicates the demand for nutritional support strategies and treatment that is individualized to the specific needs of patients [7].

## 5. Cancer-Associated Cachexia—Diagnostic Path

Diagnostic criteria for cachexia are the loss of more than 5% of weight during a 6-month period or body mass index (BMI) lower than 20 kg/m^2^ or loss of body weight more than 2% with associated sarcopenia [2,3,5,28]. Kays et al. conducted research that questions these criteria. Based on computer tomography analysis, they found that, in the studied group of patients with PC, a tissue loss of more than 5% was found in 81% of patients, but, according to traditional criteria, only 57% of them were identified as patients with cachexia. This suggests that there should be a more precise assessment of body composition in patients with cancer to provide faster detection of the wasting syndrome [29].

In routine laboratory practice, numerous biomarkers are utilized to support the diagnosis of cachexia and malnutrition. Traditionally, recommended assessments for evaluating a patient’s nutritional status included measurements of total protein, serum albumin, prealbumin, transferrin, complete blood count, electrolytes, and micronutrient levels. Possible biomarkers that can identify the early stages or risk of developing cachexia could be branched-chain amino acids (BCAA), which are markers of protein breakdown and were detected in patients with PC [29,30]. Recent studies found that evidence of the liver’s acute phase response can be detected long before weight loss and development of CAC is observed. For some cases, the levels of circulating C-reactive protein (CRP) are elevated and show a strong correlation with functional impairment. Likewise, an elevated level of NLR is associated with early oncogenesis. Neutrophilia can play an adaptive role in metabolic homeostasis during cancer progression [1]. In patients with pancreatic cancer who develop CAC, those biomarkers play a particularly important role. Many of them are not only indicators of the cachectic state but are also actively produced by PC cells themselves. This creates a self-perpetuating inflammatory loop that accelerates metabolic dysfunction and tissue degradation. Among these biomarkers, key mediators include TNF-α and pro-inflammatory interleukins such as IL-1, IL-6, and IL-8 [11]. However, recent guidelines emphasize that visceral proteins, such as albumin, are negative acute-phase reactants, primarily reflecting systemic inflammation and disease severity rather than nutritional intake alone [1,30].

Consequently, modern assessments are shifting towards consensus frameworks (e.g., The Global Leadership Initiative on Malnutrition (GLIM) criteria) [3] and composite inflammation-based scores, such as the modified Glasgow Prognostic Score (mGPS) and the CRP-to-albumin ratio (CAR), which offer higher prognostic value [5]. Furthermore, cutting-edge research advocates for the incorporation of specific biomarkers into routine analysis, including growth differentiation factor 15 (GDF-15)—a key driver of anorexia [4]—and the creatinine-to-cystatin C ratio, serving as a possibility to assess sarcopenia [6], alongside established markers like IL-6 and IGF-1 [31,32]. However, the interpretation of these results must always be based on clinical findings to minimize the risk of misdiagnosis. Some of the mentioned proteins also function as acute-phase reactants, which may not accurately reflect the true degree of malnutrition in the patient. Therefore, laboratory tests alone do not represent the gold standard for diagnosing cachexia, and the use of validated nutritional screening tools is essential [32,33]. Nevertheless, laboratory results can serve as an important complement to clinical assessment and can assist in evaluating the overall condition of the patient [31,32,33,34].

Clinical practice standards clearly specify that screening for malnutrition should be conducted at an early stage of cancer. Importantly, from a methodological perspective, such screening should be rapid, inexpensive, and highly sensitive in order to identify the risk of developing malnutrition. They must be validated, standardized, non-invasive and easy to apply in routine practice. Furthermore, clinical guidelines developed by the Oncology Evidence-Based Nutrition Practice Guideline for Adults recommend that all patients with cancer undergo malnutrition screening, and, if a risk is detected, a comprehensive nutritional assessment should be performed [34,35]. It is very important that they allow to not only identify malnourished patients but also the ones at risk. However, to date, no screening tool has been stated as a gold standard yet. The European Society for Clinical Nutrition and Metabolism (ESPEN) recommends the following screening tools: Mini Nutrition Assessment (MNA), Malnutrition Universal Screening Tool (MUST), Malnutrition Screening Tool (MST), and Nutritional Risk Screening 2002 (NRS 2002). Common parameters among all tools include unintentional weight loss and food intake. However, each tool also incorporates additional criteria that define its specific purpose. The mentioned parameters are presented below [35,36].

MNA was firstly established in the early 1990s, and it was designed to evaluate the nutritional condition of older people who may be physically weak, residing in nursing homes or staying in hospitals [37]. The full version of MNA consists of 18 items grouped into four main sections: anthropometric measurements, general health, dietary intake, and subjective self-assessment. What distinguishes it from other screening tools is the assessment of neuropsychological aspects such as dementia and depression. Since the full version of this tool is time-consuming (about 15 min), a shorter form was developed, reducing the number of items from 18 to 6. As a result, it takes only about 4 min to assess the patient’s nutritional status. Nevertheless, due to the detailed information needed to fill out the form, the participation of a trained healthcare provider is recommended. The total score ranges from 0 to 30 for the complete MNA and from 0 to 14 for the short form. A score between 17 and 23.5 signifies a potential risk of malnutrition, whereas a score under 17 on the full version or 11 and below on the short version indicates actual malnutrition. Among patients with cancer, 51% were at risk of malnutrition according to the MNA assessment [38], with patients with pancreatic cancer among the group with the highest prevalence of malnutrition. The validity of the full version as well as the short form of the MNS has been confirmed through independent clinical evaluations conducted by trained healthcare professionals and detailed assessments involving dietary intake, biochemical indicators, and anthropometric measurements [36,38].

MUST was developed in 1992, and it is widely used nowadays. MUST consists of five steps that are used to identify patients at nutritional risk and to estimate their clinical outcomes. In the first three steps, it is necessary to obtain parameters such as body mass index (BMI), the percentage of unintentional weight loss, and an assessment of the acute disease effect. The latter takes into account patients who are acutely ill and have had, or are expected to have, no nutritional intake for more than five days. Subsequently, each parameter should be scored according to the provided tables, where every component is assigned a value ranging from 0 to 2. The sum of points gives us the general risk of malnutrition, where the result of 2 or more indicates a high malnutrition risk. The fifth step involves developing a care plan and management strategy based on established guidelines. For example, patients classified as being at high risk should receive treatment that is closely monitored, and their care plan should be reviewed systematically. The ESPEN Society recommends MUST’s use for nutritional screening in outpatient settings, and its validity has been confirmed across various healthcare environments and populations [36,38,39].

MST was developed by Ferguson in 1999. It is widely used mainly due to its simplicity. MST is a brief screening instrument comprising three questions that assess unintentional weight loss, dietary intake, and appetite. The maximum possible score is 5; however, a score equal to or greater than 2 identifies a patient at risk of malnutrition, which demands appropriate actions. MST has been extensively validated in both inpatient and outpatient settings, demonstrating its reliability and practicality in diverse clinical contexts (Table 2 presents the MST form) [36,40].

NRS 2002 was developed in 2003 by Jens Kondrup’s group. This tool was derived from an analysis of 128 studies evaluating the effectiveness of nutritional support to identify malnourished patients whose nutritional status could be reversed by nutritional support. Therefore, NRS 2002 is dedicated for hospitalized patients due to its capability of detecting the risk of malnutrition. The process consists of two phases: a preliminary phase and a screening phase. The initial stage begins with four initial questions concerning whether the patient has a BMI below 20.5 kg/m^2^, has experienced weight loss within the past three months, has had a reduced food intake over the previous week, or is suffering from a severe illness. If any of the responses is “yes”, the full screening should then be conducted. It includes the evaluation of the nutritional status (BMI, weight loss, food intake) and the severity of the disease. The screening phase of NRS 2002 is summarized in Table 3. Both criteria are rated from 0 (absent score/normal) to 3 (severe), and an additional point is added to every patient aged ≥70 years old. Patients obtaining a score of 3 or higher are considered to be at nutritional risk, indicating that they may benefit from nutritional intervention and experience better clinical outcomes. The high scores of the NRS 2002 tool are associated with a longer length of hospital stay (LOS) [41], higher mortality and worse clinical outcomes for patients with cancer [36,38,40].

Other useful diagnostic tools are NUTRISCORE and GLIM [35,36,37,38]. The first one was developed by the Catalan Institute of Oncology and subsequently validated against established screening tools, such as PG-SGA (Patient-Generated Subjective Global Assessment) and MST. What distinguishes NUTRISCORE is that it was designed specifically for patients with cancer. Therefore, besides common criteria such as unintentional weight loss or evaluation of food intake, it concerns tumor location and assesses the received oncology treatment as well. The overall score, obtained by summing all categories, ranges from 0 to 11 points, with a total score of 5 or higher indicating the need for intervention, such as referral to a dietician. However, when using PG-SGA as the reference standard, NUTRISCORE demonstrated very low sensitivity in detecting malnutrition among patients with cancer in a Chinese population compared with MST [35,36,40,42].

GLIM represents a relatively recent diagnostic framework, established in 2016, yet it has become widely recognized and internationally accepted. This framework is intended for patients who have already been identified as being at risk of malnutrition, meaning that a positive result from a validated nutrition screening tool (e.g., NRS-2002 ≥ 3, MUST ≥ 1, or MNA-SF ≤ 11) is required prior to its application. It includes five categories: the evaluation of weight loss, BMI, muscle mass, food intake and inflammation. The diagnostic process involves two primary steps, corresponding to phenotypic and etiologic criteria (Table 4). To establish a diagnosis of malnutrition using this approach, the patient must meet at least one criterion from each of these two categories. It has been proven that a positive result is associated with decreased survival, particularly in elder patients with cancer [43]. In 2025, Wang et al. conducted a study evaluating the use of the GLIM criteria in comparison with the PG-SGA tool for assessing malnutrition in patients with pancreatic cancer. The study confirmed a high level of agreement between GLIM and PG-SGA in diagnosing malnutrition within this patient population. The research also demonstrated the practical advantages of GLIM, as completing this assessment requires only approximately 5 min per patient compared with around 20 min for PG-SGA [42,44]. The GLIM algorithm can be used in diverse clinical settings worldwide, including hospital inpatients, community healthcare, and the management of chronic diseases [3,26]. All mentioned screening tools are compared in Table 5.

In conclusion, there is currently no single gold standard for the early detection of cachexia associated with pancreatic cancer [9]. The diagnostic approach is consistent with the latest ESPEN recommendations, which combine nutritional, anthropometric (e.g., computed tomography), functional, and biochemical assessments [18]. Among patients with PC and CAC, GLIM and PG SGA criteria are reliable tools for identifying malnutrition, detecting functional impairments, and predicting adverse outcomes [44]. However, it should be noted that each screening tool has its strengths and weaknesses. Rapid tools such as NRS 2002, MUST, and MST are easy to use but do not quantify muscle loss, limiting the early detection of sarcopenia [11]. PG SGA offers a comprehensive nutritional profile but can be time-consuming in busy clinics [44]. Regarding GLIM, it improves assessment by integrating phenotypic (weight loss, low BMI, reduced muscle mass) and etiological (inflammation, reduced dietary intake) domains but still depends on the accurate measurement of muscle mass, for which computed tomography (CT) remains the gold standard and DXA provides a lower radiation alternative [46]. Biomarkers such as GDF-15, IL-6, CRP, and albumin offer promise for early detection, although the current evidence is limited by small cohorts and a lack of standardized cut-off points [8,47,48]. Integrating these biomarkers with established diagnostic criteria could increase the sensitivity of malnutrition and cachexia detection. Future studies combining clinical, biochemical and molecular approaches could significantly improve both our understanding of cachexia and enable its timely detection, ultimately improving patient outcomes [9].

Artificial intelligence (AI) is progressively being applied to the early identification of cachexia and pre-cachexia in oncologic patients [49]. Supervised machine learning algorithms trained on routinely collected clinical variables, such as changes in appetite, arm circumference, high-density lipoprotein (HDL) concentration, and CAR, have reported area under the receiver operating characteristic curves (AUCs) of 0.830 for cachexia and 0.701 for pre-cachexia [50]. These models exploit standard laboratory results and symptomatology, thereby offering a cost-effective screening strategy that does not require additional investigations. Explainability techniques, notably SHapley Additive exPlanations (SHAP), have been incorporated into decision support systems for lung cancer-related cachexia. Systematic reviews corroborate the high diagnostic performance of AI-based approaches (AUC > 0.80), which frequently surpass conventional screening tools when implemented with ensemble methods such as random forests or support vector machines [51]. Beyond tabular data, deep learning frameworks applied to positron emission tomography/computed tomography (PET/CT) images achieve Dice similarity coefficients of 0.92–0.94 for skeletal muscle and adipose tissue segmentation, enabling precise longitudinal monitoring of body composition changes [49]. Virtual dietitian platforms powered by AI have been shown to increase dietary adherence to 84% and to reduce hospitalization rates. Complementary logistic regression models with nomograms provide rapid risk stratification using grip strength measurements and serum creatinine levels for pre-cachexia detection [50]. While explainable AI (XAI) validates algorithmic decisions, its integration must be harmonized with physicians’ domain expertise. Furthermore, AI-driven optimization of dietitian referrals shortens waiting periods by an average of 2.4 days and facilitates personalized nutrition plans. In summary, AI enhances oncologic nutrition management and patient outcomes; however, widespread adoption necessitates methodological standardization and validation in larger multicenter cohorts [49].

## 6. Cancer-Associated Cachexia—Management

Because CAC is developed by multiple mechanisms, to either treat or prevent cachexia, there must be an applied multimodal approach. The best chance for successful therapy is a combination of nutritional, physical and pharmacological treatment. Due to the high incidence of cachexia in patients with PC, a lot of clinical trials are based on the data about their reaction to treatment [1,7]. In order to investigate whether the studies presented below that are not based on patients with PC can also be applied to them, more trials are needed.

**Surgical interventions.** Malnutrition is associated with increased postoperative complications following pancreatic surgery. CAC is a critical pathophysiological state in the surgical management of patients, a fundamental biological factor that determines both the resectability of the tumor and the physiological feasibility of major surgical intervention [52,53,54]. Although imaging findings may indicate a technically resectable tumor, the presence of cachexia frequently reflects an increased surgical risk associated with the patient’s overall physiological condition. CAC profoundly influences surgical decision making in patients with PC. Cachexia, manifested by sarcopenia, is a predictor of poor surgical outcomes [54]. Preoperative weight loss ≥ 6% was associated with significantly poorer recurrence-free survival (RFS; 8.7 vs. 17.8 months, *p* = 0.004) and overall survival (OS; 18.1 vs. 45.2 months, *p* = 0.002) of patients with PC [55]. The systemic inflammatory response associated with CAC, characterized by elevated levels of CRP and pro-inflammatory cytokines such as IL-6 and TNF-α, impairs immune function and deteriorates overall patient condition. Higher incidences of postoperative pancreatic fistula (POPF) and surgical site infections have been reported in these patients, accompanied by a significantly increased mortality rate, compared with non-cachectic individuals. Preoperative CAC markedly worsens postoperative prognosis in patients undergoing surgery for hepatopancreatobiliary malignancies, leading to a significant reduction in OS [56]. Patients with CAC are less likely to complete postoperative chemotherapy, which is essential for long-term survival in PDAC [57,58]. This complex clinical syndrome, characterized by systemic inflammation, metabolic disturbances and progressive skeletal muscle wasting, plays a crucial role in optimizing both perioperative outcomes and long-term survival.

MUST or NRS-2002, together with assessments of muscle mass via CT-based body composition analysis, BMI and inflammatory markers such as the Glasgow prognostic score, serve as important predictors of surgical risk. The decision to proceed with pancreaticoduodenectomy in a cachectic patient requires careful evaluation of the patient’s ability to withstand the metabolic stress associated with major surgery. Recent evidence indicates that preoperative CAC remains a critical determinant of postoperative outcomes, with a significantly increased risk of severe complications among patients with cancer [1,57,59]. Clinical data indicate that patients with weight loss greater than 10% of their baseline body weight exhibit significantly lower resection rates [60].

In patients with nutritional deficiencies corresponding to the pre-cachexia stage, surgical intervention is often postponed, allowing for intensive nutritional support and physical rehabilitation aimed at improving metabolic reserves [59,61]. In contrast, in cases of refractory (resistant) cachexia, where metabolic abnormalities are irreversible, surgical strategies should shift from radical resection toward palliative or less invasive approaches that prioritize QOL. Integrating nutritional status and body composition assessment into surgical decision making is crucial for minimizing operative risk and tailoring treatment to the patient’s systemic biological condition. Patients with advanced CAC demonstrate higher rates of postoperative complications, prolonged hospitalization, delayed functional recovery and limited ability to return to preoperative performance levels. Protein–energy malnutrition delays tissue repair, thereby increasing the risk of wound dehiscence and surgical site infections. The catabolic state also suppresses immune function, heightening susceptibility to postoperative infections and sepsis. Muscle wasting and loss of strength result in impaired mobility as well as diminished muscular and organ function. Patients with CAC frequently demonstrate treatment-related adverse effects to oncological therapies. In clinical practice, proactive nutritional screening and preoperative rehabilitation represent essential strategies to mitigate these risks and improve surgical outcomes [60,61,62].

Nutritional support is crucial for any surgical patient according to ESPEN guidelines. Additionally, it should control systemic inflammation and mitigate organ dysfunction. Early oral feeding is preferred for surgical patients. However, nutritional therapy should be started as soon as a nutritional risk occurs. Recommendations point to the importance of avoidance of long periods of preoperative fasting, importance of metabolic control, e.g., of blood glucose, reduction in factors that exacerbate stress-related catabolism or impair gastrointestinal function, limited period of taking paralytic agents in the postoperative period, and early mobilization to facilitate protein synthesis and muscle function. Pancreatic exocrine insufficiency (PEI) should be based on a global assessment of symptoms, nutritional status, and a pancreatic enzyme secretion. Pancreatic enzyme replacement therapy (PERT) and nutrition support need to be implemented for patients with PEI for patients after pancreatic surgery [63,64,65,66,67,68].

**Pharmacological interventions.** In Europe and the United States of America, there is no official pharmacological treatment for patients with CAC. Recommended in the latest guideline was olanzapine at a low dose, which can improve appetite and increase weight. Olanzapine is a second-generation thienobenzodiazepine antipsychotic, which is used in schizophrenia and bipolar disorder. It has beneficial influences for subjects with anorexia. For patients with CAC, it works to improve not only weight but also appetite and nutritional parameters [4,5].

The first step to find appropriate treatments is through clinical trials. Phase III-conducted studies consistently demonstrated improvements in appetite and body mass but failed to show meaningful benefits in key regulatory endpoints such as muscle strength, QOL or OS—criteria required for approval in both Europe and the United States. These limitations form the central challenge across cachexia drug development. Despite numerous clinical trials, no pharmacologic agent has yet been approved for managing CAC. Phase II trials have produced mixed results, often showing comparable appetite gains but lacking sustained effects on muscle function or survival, preventing a transition to phase III. Even agents with “positive” outcomes, such as ponsegromab or enobosarm, ultimately failed to meet regulatory standards for clinically meaningful functional improvement beyond weight increase. These shortcomings likely stem from historical technological limitations, inconsistent CAC definitions, and the use of surrogate, rather than functional, endpoints. In Japan, anamorelin achieved conditional approval despite marginal efficacy and an uncertain safety profile. To bridge these evidence gaps, future studies should prioritize multimodal phase III designs focused on functional outcomes, standardized assessment tools, and validation in diverse real-world patient populations.

One of the first clinical trials was performed by Nelson in 1994 [69], which was a phase II study of delta-9-tetrahydrocannabinol (THC) on patients with incurable cancer with a life expectancy greater than four weeks. THC acts as an orexigenic agent; it was assumed that it can help with loss of appetite. Results of the trial were promising, as THC turned out to be an effective appetite stimulant and was well-tolerated at low doses, but further studies are still needed [69]. In 1999, Loprinzi et al. conducted a phase III study in which they compared the impact of corticosteroid (dexamethasone), synthetic testosterone (fluoxymesterone) and synthetic progesterone (megestrol acetate) as a treatment for CAC. Patients were divided into groups, each receiving one of three treatment options. Then, every month there was an evaluation in terms of changes in appetite and drug toxicities. The study’s results showed that treatment with megestrol acetate was the most effective of the three treatment options in terms of the studied factors [70]. However, due to the limitations of the technology available at the time, this research is primarily of historical interest. One of the first studies in which the study group included patients with PC was published by Barber and Faeron. It was a phase I study that consisted of five patients, and the studied anticancer treatment was eicosapentaenoic acid diester (EPA). Patients were evaluated at the beginning of the study, and then every 4 weeks. The dose was controlled depending on the side effects, like sensation of fullness, cramping or nausea. They were also counteracted by the supplementation of pancreatic enzymes. The consensus of the trial was that EPA can be taken in bigger doses (around 18 g) and should be researched further [71]. In 2006 Fearon et al. presented a phase III, double-blind, and placebo-controlled trial about the anti-cachectic effects of EPA. In the study, there were 518 patients with weight loss and advanced gastrointestinal or lung cancer. Patients were randomly assigned to receive 2 g or 4 g of EPA or a placebo and assessed at 4 and 8 weeks. There was no statistically significant improvement after the treatment, which was in concordance with previous information that the supplementation of EPA-EE does not significantly inhibit lipolysis or lipid oxidation in weight-losing patients with cancer. Because of that there was no indication for approval of EPA as monotherapy in CAC [72]. Early clinical investigations demonstrated modest improvements in appetite or body mass but failed to produce consistent gains in muscle strength or QOL, which are endpoints essential for regulatory approval in Europe and the US.

Due to the very high demand for finding appropriate treatments for CAC, drug research covers not only patients with PC. In 2016, the results of two larger clinical trials were published. In the first one (NCT00467844), Crawford et al. described the phase III clinical development of the selective androgen receptor modulator (SARM) called enobosarm. The study included 300 participants consisting of non-obese men under 45 years of age and postmenopausal women with cancer. Both groups were randomly assigned to receive either a placebo or 3 mg of enobosarm daily. SARM is designed to increase lean body mass (LBM) and muscle strength, without the adverse effects typical of traditional anabolic agents. Physical function, measured by stair climb power (SCP), and LBM, assessed by dual-energy X-ray absorptiometry (DCA), were evaluated after 84 and 147 days. The trial demonstrated a significant increase in total LBM; however, improvements in SCP did not consistently reach the predefined 10% threshold that is considered a clinically meaningful functional response. OS was analyzed as a secondary safety endpoint. Although these findings suggest anabolic potential, the lack of robust evidence linking LBM gains to functional or survival benefits limited regulatory progress. Similar to anamorelin, enobosarm failed to satisfy the FDA’s requirement for demonstrable improvement in physical function, underscoring the gap between body composition endpoints and meaningful clinical outcomes in cachexia research [73]. The second trial presented by Temel et al. (NCT00219817 and NCT00267358) investigated the use of anamorelin, a ghrelin receptor agonist, which in this case was administered to patients with non-small-cell lung cancer and cachexia. Participants were randomly assigned to groups at a ratio of 2:1 to receive anamorelin once a day at a dose of 100 mg or to receive a placebo. LBM and handgrip strength were assessed over a 12-week period. LBM was increased, but handgrip strength did not change. This discrepancy limited conclusions about the functional benefit of the treatment, which remains the key determinant in cachexia therapy evaluation. Phase III of the study is currently active [74]. Accordingly, despite positive effects on body composition reported, anamorelin failed to gain FDA approval due to the lack of improvement in muscle strength and OS. Furthermore, in patients with pancreatic cancer and poor performance status, the drug did not demonstrate clinically meaningful benefits compared with those with better functional capacity. These findings underscore a key limitation of anabolic therapies in cachexia; gains in lean body mass alone may not translate into functional or prognostic improvement, which ultimately drives regulatory and clinical acceptance [75,76].

Results of a phase III, double-blind, and placebo-controlled randomized trial of mirtazapine were published in 2021. Mirtazapine is a serotonergic noradrenergic antidepressant, recommended for patients with cancer to manage some of the symptoms. It also stimulates appetite and weight gain. Hunter et al. conducted a study to assess the efficacy and tolerability of mirtazapine in the treatment of CAC. Assessed were 120 patients with incurable solid tumors and anorexia, cachexia and depression. Randomization was at a ratio of 1:1 to either receive a 15 mg dose of mirtazapine at night or a placebo for 8 weeks. The most important endpoint was change in appetite, but the trial also included changes in QOL, presence of fatigue, body weight, handgrip strength, inflammatory markers and OS. Appetite increased significantly in both the mirtazapine and placebo groups, with no difference between groups. For this reason, it has not been approved as a treatment for CAC [77]. Additionally, according to a clinical trial in 2008, mirtazapine has been used in clinical practice as an effective treatment for patients with depression and is also responsible for improving nausea, sleep disturbance, pain, insomnia and QOL of patients with cancer [78]. Although mirtazapine showed some potential to improve appetite and alleviate cancer-related symptoms, the absence of measurable benefits in muscle strength, body weight or QOL placed it among several agents that demonstrated symptomatic relief without meeting the functional and survival criteria required for regulatory approval.

Blum in 2022 [79], published results of a phase II clinical trial (NCT01127386), where the impact of the immune modulator (lenalidomide) on patients with solid malignancies who also had a concentration of C-reactive protein above 30 mg/L and cachexia was assessed. Patients received treatment in a dose of 25 mg once daily, a C-reactive protein-guided dose once daily or a placebo. The clinical trial lasted 8 weeks, and there was no significant treatment response on muscle mass and strength, therefore the trial was ended due to the need to focus on better therapeutic options [79]. Despite targeting inflammatory pathways thought to contribute to CAC, lenalidomide failed to produce measurable improvements in muscle mass or strength. It reflects the broader pattern seen in other phase II and III studies, that is, biological activity without clinically meaningful functional benefits for therapeutic advancement.

In 2022, two double-blind, placebo-controlled, and randomized trials about the efficacy of curcumin on treating CAC were also published. In the case of animal models and in vitro, it was observed that curcumin has anti-inflammatory effects by inhibiting the NF-kB signal pathway [80,81]. Chaiworramukkul et al. conducted a phase II study on 33 patients, randomized at a ratio of 1:1; the first group received 800 mg curcumin twice daily and the second group a placebo for 8 weeks. In conclusion, in the study no significant differences were found between the curcumin and placebo groups in terms of body composition. In the case of handgrip muscle strength and basal metabolic rate, clinical benefits were found [80]. Thambamroong et al.’s phase IIa study (NCT04208334) consisted of 20 patients with head and neck cancer, randomized at a ratio of 1:1. One of the groups received a placebo and the second 4000 mg of curcumin daily. There was assessment of muscle mass, body fat mass, basal metabolic rate, handgrip muscle strength, body mass index, absolute lymphocyte count, safety and toxicity of the treatment. In the study, the result was a significant increase in muscle mass compared with standard nutritional support [81]. In both cases the need for further research was highlighted.

Preclinical and clinical studies have reported anticancer effects of curcumin in pancreatic cancer, both alone and in combination with agents such as gemcitabine, 5-fluorouracil, and oxaliplatin. This therapy significantly improves functioning-associated QOL. The benefits of curcumin include its effect on heart disease, depression, and fatigue. The first to publish about its effect on PC were Li et al. Their paper presented the therapeutic effects of a curcumin (1 g/kg) and gemcitabine (25 mg/kg) mix that had a suppressing effect on tumor growth. Curcumin can also increase the effects of cisplatin, oxaliplatin and S-fluorouracil, as shown in the studies published by Duarte et al., Howells et al. and Tsai et al. Studies by Ali et al. and Soubani et al. showed changes in microRNA expression levels and anticancer effects; for example, it upregulates the expression of miR-200 and downregulates the expression of miR-21 [82]. While curcumin demonstrated anti-inflammatory and anticancer potential in both preclinical and early clinical studies, its modest or inconsistent effects on muscle strength, body composition and QOL positioned it within the broader trend of promising agents that improve biochemical or symptomatic parameters but fall short of achieving clinically meaningful endpoints required for cachexia drug approval.

In the case of patients with CAC, there is an increased level of GDF-15. Groarke in 2024 [4,5], published the outcomes of a clinical trial (ID: NCT05546476) on ponsegromab. It lasted 12 weeks, involving 187 patients. The inclusion criteria were the presence of diagnosed CAC and an elevated GDF-15 level (≥1500 pg/mL). Patients were randomized into four groups at a 1:1:1:1 ratio, and received 100 mg, 200 mg, 400 mg or placebo once every 4 weeks. Ponsegromab is a monoclonal antibody that binds circulating GDF-15, which can in theory inhibit the pathway. The endpoint was a change in body weight and improvement in appetite and cachexia symptoms. The role of GDF-15 in the development of CAC was confirmed due to the fact that treatment with ponsegromab resulted in weight gain and reduced symptoms of CAC, for example, overall activity, functionality and QOL [4,5]. New trials (NCT04725474, NCT04803305) are investigating whether blocking GDF-15 can be combined with SARM-based anabolic support to simultaneously increase appetite and muscle quality. Although ponsegromab demonstrated encouraging results in terms of weight gain and symptoms relief, its long-term efficacy in improving muscle strength, functional capacity and survival remains unproven.

Chowdhury et al. presented results of the phase II, randomized, and double-blind trial of differences between treatments of CAC with mirtazapine and megestrol. Mirtazapine is a tetracyclic antidepressant. In the trial, 80 selected patients were enrolled; some patients received 15 mg of mirtazapine daily for 8 weeks, and the control group received 160 mg of megestrol acetate daily for 8 weeks. Both drugs have similar effects on patients with CAC and can be used interchangeably. In both groups there was a noticeable improvement in CAC symptoms, like weight gain and appetite, suggesting mirtazapine as a viable alternative, particularly for those with comorbid depression [83]. This trial highlights that both mirtazapine and megestrol effectively improve appetite and body weight in CAC, yet their equivalence in symptomatic relief does not automatically resolve the broader regulatory challenge; mainly it is the lack of robust evidence that these appetite-modifying agents meaningfully improve muscle strength, physical performance or survival.

Anamorelin is approved in Japan for the treatment of CAC in certain cancer types, despite the still small amount of evidence for its effects. Yanagimoto et al. conducted an observational study with 229 patients with gastric cancer and CAC who received anamorelin from 2021 to 2023. The primary endpoint was the change of body weight at the 12 weeks mark but also assessed were appetite and food intake. The 12-week follow-up was completed by 126 patients, the median for taking the treatment was 62 days, 41% of patients completed the treatment, and 59% stopped, mainly because of disease progression. The conclusions of the study were as follows: an overall increase in body weight and lean body mass (LBM), improved appetite, and reduced systemic inflammation [84]. However, no significant changes in QOL or gut microbiota diversity were detected, and because of that and the insufficient safety data, it is currently not classified as a drug for CAC outside of Japan [83,85].

Cumulatively, these trials illustrate that multiple pharmacologic agents, from THC and megestrol to mirtazapine, EPA and newer targeted agents such as ponsegromab, can positively influence appetite, body weight or selected biomarkers, yet they repeatedly fail to demonstrate robust, consistent improvements in muscle strength, physical function or QOL, which remains the key regulatory and clinical obstacle for the approval of a dedicated anti-CAC therapy.

**Nutritional support.** Although no effective pharmaceutical cure for cancer cachexia is currently available, nutritional intervention is recommended as a fundamental component of multidisciplinary therapeutic strategy [86,87,88,89,90]. The cachexia–anorexia syndrome is complex, involving nutrient malabsorption and profound metabolic and microbiome alterations [91]. The syndrome is driven by a systemic inflammatory response mediated by pro-inflammatory cytokines. A hypermetabolic state characterized by increased catabolism, increased energy demand caused by enormous energy expenditure, protein malnutrition and anabolic potential resulting from reduced food intake, a qualitatively unbalanced diet, and pancreatic cancer pathogenesis is a significant threat to the survival of patients with pancreatic cancer [35].

Although diet is a key component of CAC management, nutritional intervention alone cannot reverse cachexia. The focus is on the inhibition of weight loss and helping to preserve muscle mass. An appropriately selected diet for the needs of the patient can have a positive impact on QOL, even improving tolerance to treatments and reducing side effects. ESPEN emphasizes that nutrition alone is often insufficient to reverse cachexia, as the underlying inflammatory and metabolic alterations driving catabolism cannot be overcome by nutritional support alone. Therefore, effective management requires addressing inflammation and metabolic dysregulation in addition to ensuring adequate nutrient intake. Interventions should occur as early as possible, before irreversible metabolic derangements at the “pre-cachexia” stage. ESPEN prioritizes achieving a total protein intake of 1.5–2.0 g/kg/day high-quality complete protein sources for those at the risk of cachexia or undergoing intensive oncological treatments. It is crucial to support muscle protein synthesis. and counteract cachexia-induced muscle wasting and systemic catabolism. ESPEN guidelines do not provide specific individualized dosage recommendations for vitamins, amino acids, or omega-3 as a standard-of-care but focus on the total nutritional support.

The primary goal of nutritional support for patients with pancreatic cancer cachexia is to counteract EPI, negative energy balance and protein breakdown, and other cachexia symptoms negatively impacting the efficacy of anticancer therapy, complications and site effects of cancer treatment [92]. Maintaining good nutritional status is a significant clinical challenge; it prevents progressive weight loss and progression of sarcopenia, is important for preserving muscle mass and alleviating asthenia, has a beneficial effect on appetite, and alleviates vitamin and nutrient deficiencies [93,94]. It also promotes an increase in both fat and lean tissue [95,96]. Omega-3 fatty acids reduce inflammation by inhibiting the production of pro-inflammatory cytokines and catabolic factors that contribute to muscle protein degradation and improve skeletal muscle maintenance. While the current evidence is insufficient to support a recommendation for omega-3 fatty acids in cachectic patients, their application as a caloric source in cancer-associated cachexia remains justifiable. Beyond caloric provision, these fatty acids may improve body weight, lean body mass, and OS [97,98,99,100,101,102,103,104,105,106,107,108].

Patients with PC who are diagnosed with PEI and exhibit symptoms or nutritional deficiencies due to maldigestion or malabsorption should receive pancreatic enzyme replacement therapy (PERT). PERT is formulation of pancreatic enzymes, according to the United European Gastroenterology and American College of Gastroenterology guidelines. Numerous studies show that fat and protein absorption increase significantly due to PERT. A decrease in EPI-related symptoms positively affects body weight and nutritional status and improves fat and fat-soluble vitamin absorption, resulting in better QOL and a reduction in mortality. However, the TriNetX analysis of the national database of 74 million patients from 54 healthcare organizations in the United States shows that only 5672 (27.3%) received PERT. Among 73,992,263 patients, 20,754 had a diagnosis of pancreatic cancer and EPI and PERT significantly improved the life of patients with PDAC [16,109,110,111]. The initial doses of PERT are recommended according to the patient’s age, severity of PEI, and fat content of the meal. The dosages for PERT are based mainly on the activity of lipase. For adult patients, an effective dose is 40,000–50,000 units of lipase with meals, and half of that dose with smaller meals, like snacks. For patients with a severe case, a higher starting dose is recommended. Results presented that PERT use was associated with lower one-year mortality (adOR 0.49), undergoing surgery (OR 0.34) and those receiving best supportive care (OR 0.12) [71]. Low doses are shown to be insufficient to avoid loss of muscle mass in patients with clinical indications of EPI during chemotherapy for PC. When using higher doses, the odds for muscle maintenance were similar to patients without EPI. OS was significantly longer in the PERT vs. non-PERT group [110,112]. Longer OS was also reported in the case of patients with metastatic PC (mPC) undergoing first-line chemotherapy with nab-Paclitaxel PERT and an ancillary diet. Also presented was an improvement in nutritional parameters, such as phase angle and free-fat mass index, after 3 months of anticancer treatment while using PERT and NS. Early and well-conducted NS in patients with mPC may impact survival and preserve performance status, thus improving QOL [113]. PERT indirectly mitigates CAC by modulating systemic inflammation and metabolic efficiency. Improved digestion supports energy homeostasis, which can promote muscle protein synthesis and attenuate the catabolic drive, increased by pro-inflammatory cytokines like IL-6 or TNF-α. In the study by Picozzi et al., 501 patients had advanced PDAC and EPI; 188 of them received PERT, which resulted in reduced weight loss (−1.5 kg vs. −2.5 kg; *p* = 0.04) and improved PNI (prognostic nutritional index), which correlated with prolonged OS (17.1 vs. 12.5 months) [112]. Management of PEI was included in 11 studies in which 78.6% of patients responded positively to PERT when it was prescribed. PEI is often overlooked as a cause of both symptoms and poor nutrition; treating it may be more common than previously [114]. This also causes gut microbiome dysbiosis, which can be returned to normal after treating it with PERT, as shown in animal studies. Gut microbiome dysbiosis has multiple downstream effects in the pancreatic modulation of the immune response and the response to chemotherapeutic agents [115]. Preliminary evidence suggests that microbiome modulation could potentially influence intestinal inflammation, enhance nutrient-sensing pathways and modulate immune responses to chemotherapy, yielding prognostic benefits for CAC [11,116,117]. Despite the benefits of PERT, it is underprescribed, mostly because of the underdiagnosis of EPI and poor interdisciplinary coordination. Routine EPI screening and early PERT initiation are recommended for PDAC supportive care by NCCN/ESMO guidelines [112,118].

Treatment of EPI is crucial in PDAC progression. It includes PERT, fat-soluble vitamin replacements, medium-chain triglycerides, and, in some cases, enteral nutrition. Nutritional optimization should be implemented promptly, following established guidelines, to improve QOL and OS [119,120]. Targeted dietary supplementation with PERT has been shown to beneficially modulate metabolism, impact the microbiome and reduce systemic inflammation [91,93,121,122]. In the study published by Ritz et al., feces of pigs were analyzed by next-generation sequencing. Data collected in this study showed that PERT can reverse EPI-induced dysbiosis to an almost healthy state. PERT causes a reduction in bacterial overgrowth and elevated α-diversity [116].

Furthermore, evidence suggests that patients with advanced PDAC and with nutritional support, pancreatic enzyme replacement therapy, and well nourishment exhibit lower complications, better tolerance to oncological treatments and experience fewer complications and toxicity-related side effects [119,123]. Moreover, the present research indicates that manipulating the microbiome can modify TME, increasing the effectiveness of existing treatments and leading to new therapies, and microbiomes can improve the effectiveness of chemotherapy. The microbiome works as a key regulator of anticancer immunity, modulating the activation of T lymphocytes, macrophage polarization, and dendritic cell function; and inhibiting or enhancing immune surveillance affecting the effectiveness of immunotherapies, immune checkpoint inhibitors (ICI), and CAR-T cell therapy. Approaches including dietary modification, probiotics, fecal microbiota transplantation (FMT) and microbial metabolite supplementation show promise in restoring and improving immune homeostasis treatment results [124]. The interventions accelerate protein turnover and enhance both carbohydrate and lipid metabolism. These metabolic alterations subsequently reshape the composition of the gastrointestinal microbiome, which in turn contributes to mitigating PDAC-associated cachexia [125].

**Gut microbiome in CAC:** The cancer-associated microbiome constitutes an enabling hallmark of cancer, exerting a profound influence on PDAC progression by modulating tumor cells, precursor lesions and the surrounding non-neoplastic cells. Dysbiosis in the intestinal microbiome of patients with PDAC impairs gut barrier integrity, facilitating microbial translocation and chronic inflammation. Furthermore, intratumoral microbiota, alongside bacterial metabolites, pro-inflammatory mediators and associated metabolic dysregulation, collectively drive oncogenesis, immune evasion and therapeutic resistance in PDAC [126,127,128,129]. Analysis of data has shown that *Pseudomonas* sp. is the predominant species detected in PC [130]. Additionally, Shannon’s index and β-diversity significantly differ between patients and the controls in saliva, fecal, and blood samples. Higher numbers of *Lactobacillus*, *Enterobacter* and *Prevotella* bacteria were observed not only in stool but also in saliva and blood samples [131]. Moreover, differences in the composition of intestinal microflora, SCFA and inflammatory parameters have been demonstrated in patients with pancreatic cancer cachexia. Proteobacteria (*p* < 0.001), Enterobacteriaceae (*p* < 0.01), *Veillonella* (*p* < 0.001), *Megamonas* (*p* < 0.05) and *Peptococcus* (*p* < 0.001) were more common among patients with cachexia. A higher abundance of *Lactobacillus* in patients with PDAC was correlated with improved progression and OS, further supporting the therapeutic potential of *L. reuteri* and showing that the supplementation of psychobiotic *Lactobacillus* may offer a novel therapeutic strategy for PDAC [132]. Lower fecal SCFA concentrations were observed in patients with cachexia. Fecal calprotectin concentration was positively correlated with the abundance of *Peptococcus*, unknown Enterobacteriaceae and *Veillonella*, as well as clinical and microbiological parameters [133].

Moreover, metagenomic analyses have revealed stage- and metastasis-dependent alterations in the intestinal microbiota composition of patients with PDAC, characterized by increased α-diversity relative to healthy controls. In non-metastatic PDAC cohorts, 59 differentially abundant microbial species were identified (56 increased), while metastatic PC cases exhibited 21 such species (19 increased), accompanied by 18 dysregulated microbial metabolic pathways. Notably *Klebsiella pneumoniae*, *Klebsiella oxytoca* and *Akkermansia muciniphila* emerged as predominant carriers of antibiotic resistance genes (ARGs) in the gut microbiota of patients with metastatic PDAC [134].

The published research demonstrate that the composition of the gut microbiome and metabolome can modulate anti-tumor immune functions, affecting the response to oncological therapy [135].

Importantly, research on the microbiome’s role in PC suggests a promising role for microbiota profiles as potential non-invasive diagnostic biomarkers. These findings point toward their future utility in early detection, therapeutic targeting, and the prediction of treatment responses or immune-related adverse events [123,128,136]. Nevertheless, clinical validation remains necessary to confirm both the diagnostic utility of microbiome analysis and its intricate interplay with pancreatic tumor metabolism. Furthermore, the potential of microbiota modulation as a strategy for the prevention and management of cachexia warrants extensive investigation in future studies and clinical trials.

**Physical exercise.** One of the ways to help with the effects of cachexia is physical exercise. Appropriate exercise affects protein synthesis, thus possibly blocking muscle loss. Movement also has a positive impact on reducing inflammation and improving QOL [7]. Psychical exercise as a part of treatment for CAC focuses on improving muscle metabolism and preserving/increasing muscle mass. Exercises stimulate protein synthesis pathways, e.g., IGF-1/Akt/mTOR, while at the same time suppressing proteolysis (e.g., autophagy), thereby blocking skeletal muscle loss. In 2023 and 2024, studies were published highlighting the fact that exercise helps to stabilize weight or even limit weight loss, increasing the strength properties of weight training. This is important because it correlates with the physical autonomy of patients, which has a positive impact on QOL. Exercise can also help with inflammation and promoting the synthesis of proteins. Regularity leads to the release of anti-inflammatory cytokines, such as IL-10, which can modulate the response of the immune system, counteracting the IL-6/TNF-α axis, which drives CAC catabolism. The overall impact on survival has inconclusive evidence and this topic needs to be better researched. Based on clinical studies, exercise should consist of two–three sessions that are matched to patients’ conditions. It can contain aerobic exercises (cycling on a stationary bike, treadmill) for 20–30 min or resistance exercises (elastic bands/body weight) twice a week. It could also combine both programs [7,137,138,139].

## 7. Conclusions and Future Directions

There is an urgent need for further research into cachexia metabolism and its management. Individualized treatment and nutritional interventions are necessary to delay the onset of cachexia symptoms or mitigate the progression of cachexia.

PDAC is closely linked to the development of cancer-associated cachexia (CAC), a multifaceted metabolic syndrome that significantly impairs quality of life and, more importantly, remains a primary determinant of morbidity and therapeutic failure. Rather than a late-stage manifestation of malignancy, CAC is a dynamically devastating systemic syndrome driven by the synergy of tumor-derived catabolic factors, persistent systemic inflammation, and profound neuroendocrine dysregulation. PDAC-related cachexia is characterized by an unbalanced metabolic rearrangement, and the unique severity of cachexia in PDAC is exocrine pancreatic insufficiency (EPI) and malabsorption, which amplify patient wasting. CAC must be differentiated from simple starvation or what is important from the progression of cancer, and it must be understood that conventional nutritional support is insufficient.

Despite significant advances in our understanding of the molecular triggers of cachexia (IL-6, TNF-α, and GDF-15), a critical translational gap persists between project or laboratory discovery and implementation in clinical practice. We currently lack validated high-sensitivity biomarkers capable of identifying the pre-cachectic state—the only stage where metabolic intervention is likely to be truly effective and reversible. Furthermore, the inherent heterogeneity of patients with cachexia or PDAC suggests that host-specific genetic predispositions and the complex interplay of the gut–pancreas axis remain inadequately tested in current clinical trial designs. Future vision: towards metabolic stabilization. PDAC management must evolve toward metabolic individual precision oncology. Successful therapy is not merely standard but should be anticipatory, using the best diagnostic tools, real-time monitoring of body composition (e.g., CT-defined sarcopenia) and metabolic profiling. The ultimate goal is to achieve metabolic stabilization through multimodal pharmacological recommendations that simultaneously target systemic inflammation, inhibit muscle wasting, and sarcopenia.

Translating the current evidence into clinical practice requires modifications to the standard of care, with individualized recommendations. Clinicians should implement pancreatic enzyme replacement therapy (PERT) early, with proper, even high-dose, supplementation at the time of diagnosis to mitigate the malabsorption–wasting problem, regardless of overt symptoms. Body composition assessment and monitoring should transcend simple weight measurements. CT imaging with metabolic profiling and diagnostic tools for assessing sarcopenia should play an essential role in accurate prognostic stratification and early intervention.

According to the current international guidelines, cachexia management must be multidisciplinary, combining nutritional optimization and structured resistance exercise to preserve functional status and improve tolerance to systemic chemotherapy.

The development of robust guidelines is essential to optimize multimodal care, improve nutritional support, and enhance quality of life—both physical and mental—for patients with cachexia.

## Figures and Tables

**Table 1:** Mechanisms of CAC pathogenesis by selected cytokines.

Cachexia Mediator	Expression Levels	Symptoms	Mechanism	Source
IL-1β	↑ in serum and muscle tissue of patients with CAC	Muscle wasting, fatigue	Increases expression of MAFbx and MuRF1, marking proteins of C2C12 myotubes for proteasomal degradation	[1,20]
IL-6	↑ serum levels, correlated with disease severity in PDAC	Systemic inflammation, weight loss, reduced survival, anorexia, fatigue	Mediates systemic inflammation by interacting with TNF-α and inducing CCL2 production; regulates signaling pathways via MuRF1 and atrogin-1 release; binds receptor to activate STAT3, stimulating myostatin and inhibiting myogenesis	[1,3,20]
CCL2	↑ in circulation and adipose tissue during cachexia	Liver inflammation, neuroinflammation, weight loss, metabolic changes in muscle	Directs CCR2-driven macrophage migration; promotes inflammation and metabolic shifts in muscle/WAT; induces IL-6 production	[1]
Myostatin	↑ circulating levels and muscle expression in advanced cancer	Lean body mass loss, muscle atrophy	Induces autophagy/proteolysis via AcvR2B activation; represses Akt/mTOR pathway, inhibiting muscle growth	[20]
Activin A	↑ serum and tumor-derived levels	Lean body mass loss, muscle atrophy	Induces autophagy/proteolysis via AcvR2B activation	[1]
TNF-α	↑ in serum, tumor microenvironment	Muscle atrophy	Activates ubiquitin ligase genes, destroying myofibrillar proteins/transcription factors; triggers E3 ligase pathway for muscle degradation	[1,20]
GDF-15	↑ markedly in serum of patients with PDAC-CAC	Anorexia, weight loss, muscle atrophy, fibrosis, reduced food intake	Influences hypothalamic/brainstem hunger centers via GFRAL receptor; activates SMAD2/3 signaling akin to myostatin, inhibiting protein synthesis/degrading it; stimulates HPA axis, elevating glucocorticoids	[1]
LMF	↑ serum levels in patients with PDAC-CAC	Lipolysis, chemotherapy resistance	Released from WAT/tumor/adipose tissue due to cytokines; drives lipolysis, lipid mobilization, WAT browning, energy dissipation; upregulates UCP-2 (linked to ROS detoxification/chemoresistance); interferes with pro-inflammatory AOC3 functions	[11]

↑ higher concentration, increase; IL-1β: interleukin 1 beta; IL-6: interleukin 6; CCL2: C-C motif chemokine ligand 2; TNF-α: tumor necrosis factor alpha; GDF-15: growth differentiation factor 15; LMF: lipid mobilizing factor; MAFbx: muscle atrophy F-box; MuRF1: muscle-specific RING finger protein 1; STAT3: signal transducer and activator of transcription 3; CCR2: C-C motif chemokine receptor 2; AcvR2B: activin receptor type-2B; GFRAL:GDNF family receptor alpha-like; HPA axis: hypothalamic–pituitary–adrenal axis; WAT: white adipose tissue; AOC3: amine oxidase copper-containing 3.

**Table 2:** Malnutrition Screening Tool (MST). Based on [40].

Have you lost weight recently without trying? No Unsure	0 2
If yes, how much weight (kilograms) have you lost? 1–5 6–10 11–15 >15 Unsure	1 2 3 4 2
Have you been eating poorly because of a decreased appetite? No Yes	0 1
Total	
A score of 2 or more identifies a patient at risk of malnutrition.	

**Table 3:** The screening phase of Nutritional Risk Screening 2002 (NRS 2002).

Impaired Nutritional Status	Severity of Disease (Stress Metabolism)
Absent (Score 0) Normal nutritional status	Absent (Score 0) Normal nutritional requirements
Mild (Score 1) Weight loss > 5% in 3 months or Food intake 50–75% of normal requirement in the preceding week	Mild (Score 1) Hip fracture Chronic patients, particularly with acute complications (e.g., cirrhosis, COPD, chronic hemodialysis, oncology, diabetes)
Moderate (Score 2) Weight loss > 5% in 2 months or BMI 18.5–20.5 kg/m^2^ + impaired general condition or Food intake 25–50% of normal requirement in the preceding week	Moderate (Score 2) Major abdominal surgery Stroke Severe pneumonia Hematologic malignancy
Severe (Score 3) Weight loss > 5% in 1 month or >15% in 3 months or BMI < 18.5 kg/m^2^ + impaired general condition or Food intake 0–25% of normal requirement in the preceding week	Severe (Score 3) Head injury Bone marrow transplantation Intensive care patients (APACHE > 10)

APACHE = acute physiology and chronic health evaluation; BMI = body mass index; COPD = chronic obstructive pulmonary disease. Based on [40,41].

**Table 4:** Overview of the GLIM diagnostic criteria, including phenotypic and etiologic categories used to identify malnutrition in at-risk patients [45].

Criteria Category	Diagnostic Components	Thresholds and Definitions	Assessment Methods
Phenotypic criteria	Weight loss (%)	>5% within past 6 months or >10% beyond 6 months	Medical records/patient recall; calibrated scales
	Low BMI (kg/m^2^)	<20 if <70 years, or <22 if ≥70 years <18.5 if <70 years, or <20 if ≥70 years (for Asian populations)	Height/weight measurement (adjust for edema)
	Reduced muscle mass	Determined by validated body composition measuring techniques, e.g., ASMI: M < 7.0 kg/m^2^; F < 5.7 kg/m^2^ (for Caucasians) ASMI/ALMI: M < 7 kg/m^2^; F < 5.4 kg/m^2^ (for Asian populations) CC: M < 33 cm; F < 32 cm	Gold standards: DXA/CT/MRI/BIA/US Other methods: anthropometry CC/MUAC
Etiologic criteria	Reduced food intake or assimilation	Energy intake < 50% of needs for >1 week Malabsorption (e.g., chronic diarrhea, IBD, SBS)	24 h dietary recall; clinical evaluation of GI function
	Inflammation	Acute: associated with major infection, e.g., sepsis, trauma, major surgery Chronic: active cancer, congestive heart failure, chronic obstructive pulmonary disease, chronic renal disease, or any disease with chronic or recurrent inflammation	Lab tests (including measurement of C-reactive protein); disease activity indices

ALMI = appendicular lean mass index; ASMI = appendicular skeletal muscle mass index; BIA = bioelectrical impedance analysis; BMI = body mass index; CC = calf circumference; CT = computed tomography; DXA = dual-energy X-ray absorptiometry; GI = gastrointestinal; IBD = inflammatory bowel disease; MRI = magnetic resonance imaging; MUAC = mid-upper arm muscle circumference; SBS = short bowel syndrome; US = ultrasound.

**Table 5:** Overview and comparison of commonly used nutritional screening and diagnostic tools, including target populations, clinical settings, parameters assessed, completion time, and consideration of disease severity. Based on [36,38,40].

Tool	Target Population	Recommended for Patients with PC?	Clinical Setting	Key Parameters Assessed	Time to Complete [min]	Includes Disease Severity?
NRS-2002	Hospitalized adults	Recommended	Hospital (inpatient)	Unintentional weight loss, BMI, food intake, disease state	3–5	Yes
MNA (Full/Short Form)	Older adults (≥65 years)	Recommended	Hospital, outpatient, long-term care	Unintentional weight loss, BMI, age, neuropsychological aspects	5–15 (depending on version)	No (not directly)
MUST	Adults	Strongly recommended	Hospital and outpatient settings	Unintentional weight loss, BMI, appetite, food intake	3–5	Partially (acute disease effect)
MST	Hospitalized adults and, outpatients	Recommended	Hospital, especially oncology units	Unintentional weight loss, appetite, food intake, muscle mass/function/mobility	1–3	No
NUTRISCORE	Patients with cancer	Requires validation in patients with PC	Oncology settings (hospital/outpatient)	Unintentional weight loss, food intake, tumor location, oncology treatment	3–5	Indirectly (oncology-related factors)
GLIM Criteria	Adults (incl. oncology)	Strongly recommended	All clinical settings	Phenotypic criteria (weight loss, BMI, reduced muscle mass); etiologic criteria (reduced food intake, inflammation)	Requires full clinical assessment	Yes (etiologic criteria)
PG-SGA Short Form	Inpatients with cancer	Strongly recommended	Ambulatory oncology settings	Unintentional weight loss, food intake, muscle mass/function/mobility, disease state	5	Yes

BMI = body mass index; GLIM = the Global Leadership Initiative on Malnutrition; MNA = Mini Nutrition Assessment; MST = Malnutrition Screening Tool; MUST = Malnutrition Universal Screening Tool; NRS-2002 = Nutritional Risk Screening 2002; PC = pancreatic cancer; PG-SGA = Patient-Generated Subjective Global Assessment.

## Data Availability

No new data were created or analyzed in this study.

## Abbreviations

The following abbreviations are used in this manuscript

GDF-15	growth differentiation factor 15
PC	pancreatic cancer
CRC	colorectal cancer
PDAC	pancreatic ductal adenocarcinoma
REE	resting energy expenditure
ESPEN	the European Society for Clinical Nutrition and Metabolism
GLIM	the Global Leadership Initiative on Malnutrition
EPI	exocrine pancreatic insufficiency
PERT	pancreatic enzyme replacement therapy
SCFA	short-chain fatty acids
CAC	cancer-associated cachexia
PTHrP	parathyroid hormone-related protein
BMI	body mass index
QOL	quality of life
OS	overall survival
TNF-α	tumor necrosis factor alpha
IFN-γ	interferon-gamma
TGF-β	transforming growth factor beta
IL	interleukin
NPY/AgRP neurons	neuropeptide Y/agouti-related peptide neurons
POMC/CART neurons	pro-opiomelanocortin/cocaine- and amphetamine-regulated transcript neurons
UPS	ubiquitin–proteasome system
LMF	lipid mobilizing factor
MAFbx	muscle atrophy F-box
MuRF1	muscle-specific RING finger
CCL2	C-C motif chemokine ligand 2
STAT3	Signal transducer and activator of transcription 3
AcvR2B	activin receptor type-2B
GFRAL	GDNF family receptor alpha-like
HPA axis	hypothalamus–pituitary–adrenal axis
WAT	white adipose tissue
AOC3	amine oxidase copper-containing 3
BCAA	branched-chain amino acids
CRP	C-reactive protein
mGPS	Modified Glasgow Prognostic Score
CAR	CRP-to-albumin ratio
ESPEN	European Society for Clinical Nutrition and Metabolism
MNA	Mini Nutrition Assessment
MUST	Malnutrition Universal Screening Tool
MST	Malnutrition Screening Tool
NRS 2002	Nutritional Risk Screening 2002
APACHE	acute physiology and chronic health evaluation
COPD	chronic obstructive pulmonary disease
PG-SGA	Patient-Generated Subjective Global Assessment
ALMI	appendicular lean mass index
ASMI	appendicular skeletal muscle mass index
BIA	bioelectrical impedance analysis
CC	calf circumference
CT	computed tomography
DXA	dual-energy X-ray absorptiometry
GI	gastrointestinal
IBD	inflammatory bowel disease
MRI	magnetic resonance imaging
MUAC	mid-upper arm muscle circumference
SBS	short bowel syndrome
US	ultrasound
AI	artificial intelligence
HDL	high-density lipoprotein
AUCs	area under the receiver operating characteristic curves
SHAP	SHapley Additive exPlanations
PET	positron emission tomography
XAI	explainable AI
THC	delta-9-tetrahydrocannabinol
EPA	eicosapentaenoic acid diester
SARM	selective androgen receptor modulator
LBM	lean body mass
SCP	stair climb power
mPC	metastatic PC
PNI	prognostic nutritional index
ICI	immune checkpoint inhibitors
FMT	fecal microbiota transplantation
